# Risk Factors for Acute Brucellosis in Patients on the Day of Admission at Selected Hospitals of Abbottabad, Pakistan

**DOI:** 10.3389/fpubh.2021.669278

**Published:** 2022-01-31

**Authors:** Laiba Hassan, Shahzad Ali, Muhammad Ali Syed, Asim Ali Shah, Shahid Ahmad Abbasi, Sadia Tabassum, Usama Saeed, Falk Melzer, Aman Ullah Khan, Hosny El-Adawy, Heinrich Neubauer

**Affiliations:** ^1^Department of Microbiology, The University of Haripur, Haripur, Pakistan; ^2^Wildlife Epidemiology and Molecular Microbiology Laboratory (One Health Research Group), Discipline of Zoology, Department of Wildlife and Ecology, University of Veterinary and Animal Sciences, Ravi Campus, Pattoki, Pakistan; ^3^Department of Microbiology, Fauji Foundation Hospital, Rawalpindi, Pakistan; ^4^Department of Zoology, Hazara University, Mansehra, Pakistan; ^5^Institute of Bacterial Infections and Zoonoses, Friedrich-Loeffler-Institut, Jena, Germany; ^6^Department of Pathobiology, College of Veterinary and Animal Sciences, Jhang, Pakistan; ^7^Faculty of Veterinary Medicine, Kafrelsheikh University, Kafr El-Sheikh, Egypt

**Keywords:** brucellosis, abortion, real time PCR, serum agglutination test, Pakistan

## Abstract

Brucellosis is a neglected zoonotic disease of ruminants. It causes severe health problems in humans and significant economic loss. Only a limited number of studies have been conducted in Pakistan to determine the prevalence of human brucellosis and related risk factors. The objectives of the current cross-sectional study were to determine the prevalence of anti-*Brucella* antibodies in sera collected from symptomatic patients at three hospitals of Abbottabad using a commercial slide agglutination test (SAT) and to determine risk factors for brucellosis for these patients. Five hundred blood samples were collected. A questionnaire was filled in for each patient to obtain information on age, gender, living area, brucellosis associated symptoms, associated risk factors, pregnancy and abortion history. A total of 13.6% (*n* = 68) patients were found to be SAT positive and in 83.3% (*n* = 57) of these samples *Brucella* DNA was detected by genus specific RT-PCR for BCSP-31 gene. Statistical analysis was performed to determine odd ratios, risk ratios, 95% confidence intervals, and *p*-values. The prevalence of brucellosis by SAT was reported to be higher in women (14.6%, *n* = 44) than in men (12.1%, *n* = 24). The age group 25–50 years was found to be at higher risk for brucellosis (14.5%, *n* = 50) “animal contact” was reported as the main risk factor followed by “consumption of raw animal products.” Out of 131 pregnant women and 21 patients had abortion, the seropositivity of Brucellosis was 9.9% and 23.8%, respectively. The present study reports a striking prevalence of brucellosis among patients including pregnant women at three hospitals of Abbottabad. These findings may foster strategies for controlling human brucellosis at household level, raising of awareness about brucellosis in hospital and family doctors, and finally in setting up an eradication program in the dairy industry.

## Introduction

Brucellosis is a disease caused by bacteria of species of the genus *Brucella* with a high zoonotic potential (1). In developing countries, the disease is of great importance for public and veterinary health (2), affecting both human and animal health (3). Endemic areas include the Mediterranean region, the Middle East, the Arab peninsula, Africa, Latin America and Asia (4). Four species of *Brucella* (*Brucella abortus, Brucella melitensis, Brucella suis, Brucella canis*) are known to cause disease also in humans regularly. Other *Brucella* species i.e., *Brucella inopinata, Brucella ceti*, and *Brucella microti* cause disease in animals, but rarely in humans (5).

The number of new *Brucella* infections in humans exceeds 500,000 cases per year worldwide (4). A limited number of reports on the seroprevalence of brucellosis in different population groups of Pakistan is available. The studies are only comparable to a limited extent as they use different techniques and study designs. The prevalence of anti-*Brucella* antibodies in patients who visited hospitals of Peshawar, Khyber Pakhtunkhwa (KPK) for routine checkup was found to be 29.9% (6). While 17 and 11% patients from hospitals of Lahore were found positive by Rose Bengal plate test (RBPT) and real-time PCR, respectively (7). High risk professionals in close contact to farm animals often had noticeable high prevalence. 18.42 and 27.47% of women from district Malakand, KPK were reported positive using serum agglutination test (SAT) and enzyme linked immonosorbent assays (ELISA), respectively (8). 32.9% of livestock farmers and 32.67% breeders were diagnosed positive using serum plate agglutination test (SPAT) in Peshawar, KPK (6). These high values are in contrast to prevalence reported in animal keepers of Charsadda, KPK, Pakistan i.e., 12.5 and 6.25% when SPAT and PCR were applied (9). A total of 21.7% slaughter house workers were diagnosed positive in Lahore (Punjab Province) by ELISA (10), while 6.9 and 5.7% of probands were positive when high risk individuals of the Potohar region were tested by real time PCR and serology (RBPT and SAT). Considerably higher prevalences in high-risk individuals from the district Faisalabad of the Punjab were noticed only several years later i.e., 38.94% by SAT and 14.17% by PCR (11). From KPK and Punjab 5.32 and 14.8% of patients with febrile illness were tested positive using SAT (12) and 10.1 and 5.8% of patients in Rawalpindi and Islamabad hospitals using RBPT and real-time PCR assays, respectively (13). Brucellosis may also be found in patients with suspected tuberculosis when using SAT (14). The detection of anti-*Brucella* antibodies from pregnant women from hospitals of the Rawalpindi region, Pakistan was also documented (15).

The main driver for human brucellosis is brucellosis of farm animals. For example, 3.97% milk and 7.94% of blood samples of cattle were diagnosed positive for brucellosis by MRT and SPAT in the districts Lakki Marwat and Bannu of KPK, Pakistan (16). Bovine brucellosis was investigated in various herds of cattle on the Potohar plateau, Pakistan by using serological and molecular techniques. So, 170 (6.3%) positive serum samples out of 2,709 (1,247 buffaloes and 1,462 cattle) were tested positive by the RBPT and 52.4% of these positive samples contained *Brucella* DNA. Furthermore, 156 (6.7%) milk samples out of 2,330 (1,162 buffaloes and 1,168 cattle) were positive by MRT (17).

In Pakistan, the most common risk factors for human brucellosis are: age, male gender, urban residence, unsafe practices during parturition of animals, raw milk consumption, contact with animals, experience of intra-uterine death, history of miscarriage contact with women who suffered miscarriage, and contact with aborted animals (7, 8, 15, 18, 19). Occupational groups at high risk of brucellosis include butchers, livestock farmers/breeders, milkers, veterinarians, inseminators, laboratory workers as well as individuals associated with packing and selling dairy products and raw meat (6, 10, 11, 15, 19).

No current test is capable of diagnosis all different stages of brucellosis (e.g., incubation, acute, chronic, relapse) alone. Although isolation is considered as the gold standard low sensitivity and high risk of infection for the personnel restricts its use to specialized laboratories (20). Thus, serological tests like SAT (RBPT and slide or plate SAT), complement fixation test (CFT) and ELISAs are used in routine diagnosis (20). Thus, a combination of different tests should be used for definite diagnosis. Usually, SAT is used for screening and Coombs' test or CFT for confirmation. Recently, real-time PCR was used to identify *Brucella* DNA in serum samples (3, 7). This technique adds evidence to the serological diagnosis but may show negative results in brucellosis patients as it depends on the presence of reasonable amounts of circulating DNA in the sample. In resource poor countries the use of rapid febrile antigen *Brucella* slide agglutination tests (SAT) is common as it is a cheap alternative to other tests. These tests are considered useful for diagnosis of acute and subacute brucellosis when patients show clinical symptoms but fail to identify chronic cases (20). Hence, it may be a valuable tool in diagnosis of fever of unknown origin caused by brucellae as genus specific PCRs exist (21, 22).

According to World Health Organization (WHO) and Organization of Animal Health (OIE), brucellosis is a neglected zoonotic disease which has a negative impact on human health and animal production (19, 23). In Pakistan, multiple zoonotic diseases (i.e., toxoplasmosis, brucellosis, etc.) are prevalent in the human population (24–26). However, shortcomings have been observed about data related to human brucellosis in KPK province of Pakistan. The present study was aimed to investigate the prevalence of acute brucellosis in symptomatic patients at the day of admission to hospitals of Abbottabad, KPK, Pakistan and to identify risk factors for *Brucella* infection in these patients.

## Materials and Methods

### Study Area

The study was carried out at Ayub Medical Hospital, Jinnah Medical Hospital, and DHQ Hospital of Abbottabad city. Abbottabad city is the capital of the Abbottabad Division located in the Hazara region, province KPK. It has a total area of about 1,967 km^2^ or 759 square miles. According to the census of 2017, the total population is 1,332,912 and the density is 680 inhabitants per km^2^ (25). The total number of households of the district reporting livestock such as cattle, buffaloes, sheep, goats and camels etc. is 1,44,207 and the total number of reported animals of the KPK province is 5,967,886 according to data of 2006 (27). Most of the rural population has to do livestock farming as there is little land available for agriculture in this district. Thus, a higher risk of acquiring brucellosis due to close contact to livestock can be supposed.

### Study Design and Collection of Patients Detail by Questionnaire

A cross-sectional study was conducted with no follow up investigation. Blood samples (*n* = 500) were randomly collected from symptomatic patients who visited the outdoor patients department (OPD) of the hospitals and agreed to take part in this study from April, 2019 to August, 2019. Ethically and professionally, neither information nor samples were collected from patients hesitant to participate in this study. Men and women included in this study were older than 20 years.

A questionnaire was filled in personally for each patient. Questions on age, gender, dwelling area, animal ownership or presence of animals in household, contact with animals, processing or handling raw animal products or meat, consumption of raw animal products, access of livestock to the household's source of drinking water, abortion in animals or contact with aborted animals, presence or previous history of symptoms such as fever, night sweats, headache, arthralgia, generalized ache, nausea, anorexia, and fatigue, and presence of such symptoms or brucellosis in any other house-hold member had to be answered. Women were asked to report on previous pregnancies and abortion history.

### Blood Collection

About 4 ml blood was collected aseptically from the brachial vein with a disposable, sterile syringe. Blood was immediately injected and transferred into serum separating gel-tubes and tubes were labeled immediately. The serum was obtained by centrifugation (Centrifuge 800, China) at 1,790 × g for 5 min. Each serum sample was divided into two parts for SAT and DNA extraction to perform RT-PCR, respectively.

### Serology

The *Brucella abortus* antigen of the Febrile Antigen Kit (Plasmatec, Lab21 Healthcare Ltd., Bridport, Dorset, United Kingdom) was used for serum agglutination slide test as per manufacturer's instructions. Briefly, 80, 40, 20, 10 and 5 μl of undiluted serum was added onto a row of 3 cm diameter circles of a reaction slide. Then a drop of the undiluted suspension of antigen was added to each serum sample using the dropper provided with the kit. The content was mixed using a stirring stick. The slide was shaken gently for 1 min and then observed for any agglutination. A test was positive when agglutination was observed at 1:80.

### DNA Extraction and Quantification

DNA was extracted from seropositive serum samples (*n* = 68) by using WizPrep gDNA Mini Kit (Wizbiosolutions Inc., Jungwon-gu, Seongnam, South Korea) according to the instructions and protocol of the kit manufacturer. After extraction of DNA from serum samples, Nanodrop-1000 UV spectrophotometer (Nano-Drop technologies, Wilmington, DE) was used for DNA quantification. DNA quantification was performed by measuring absorbance at 260 nm and DNA purity was checked with the ratio of 260/280. A value of approximately 1.8 was considered to show pure DNA. The purified DNA samples were stored at −20°C.

### Real-Time Polymerase Chain Reaction

Real-time PCR was using a MJ Mini Bio-RAD Thermal cycler (Applied Biosystems, Foster City, California, USA). Genus specific primers and probes targeting the BCSP-31 gene were used according to Probert et al. (28). The BCSP-31 gene codes for a 31 KDa immunogenic protein of the membrane and is conserved among all *Brucella* species and biovars. The sequences of primers and fluorescent tagged probe are given in the Table 1. A total of 25 μl of reaction mixture was prepared for the amplification of each sample. The reaction mixture was prepared by adding 5 μl of 5x Amplicon qPCR master mix (Solis BioDyne, Teaduspargi, Tartu, Estonia), 0.8 μl forward primer (10 pmol/μl), 0.8 μl reverse primer (10 pmol/μl), 0.4 μl probe (5 pmol/μl), 3 μl (DNA concentration range 0.26–33 ng/ml) extracted DNA sample and 15 μl of nuclease free water to a final volume of 25 μl.

**Table 1:** Sequences of probe and primers for genus specific *Brucella* RT-PCR.

**Target gene**	**Probe and primers**	**Sequences**
BCSP-31	Probe	5′-FAM-AAATCTTCCACCTTGCCCTTGCCATCA- BHQ1-3′
	Forward	5′-GCTCGGTTGCCAATATCAATGC-3′
	Reverse	5′-GGGTAAAGCGTCGCCAGAAG-3′

The PCR conditions were initial denaturation for 10 min at 95°C, 44 cycles of 20 s at 95°C for denaturation, 50 s at 60°C for primer annealing and 50 s at 72°C for DNA extension. The results were considered positive when the cutoff value was <40 cycles.

### Statistical Analysis

For the data analysis, a patient was considered positive if she/he had positive SAT result. Data were statistically analyzed by using the online tools of Vassar Stats (Vassar College; Poughkeepsie, NY USA; http://vassarstats.net/). The VassarStats is a useful and user-friendly tool for performing basic statistical computations such as basic probabilities, correlation and regression, *t*-tests, and procedures, proportions, simulations, properties of normal distribution and analysis of covariance. The site includes a helpful table on the platforms and browsers needed to run particular simulations, and each page provides examples to key concepts. Collected data and results were categorized into groups. Version 2 software was used for analysis of logistic regression to determine odd ratio, risk ratio, 95% confidence interval and Chi-square test for *p*-Value. Fisher exact test was used in case when the cross table had five or less counts. The data were considered to be statistically significant with a *p*-value ≤0.05.

## Results

Out of 500 samples, 68 samples were found to be SAT positive. From 57 out of these 68 seropositive samples *Brucella* DNA sequence was amplified by real-time PCR (Table 2). The associations of demographic factors with seropositivity for *anti-Brucella* antibodies are given in Table 2. The study showed that the prevalence of brucellosis was higher in the age group 25–50 years (*n* = 50). The prevalence of brucellosis was 12.1% (*n* = 24) in males and 14.6% (*n* = 44) in females but this finding was not significant (*p* = 0.493). The prevalence of disease was reported to be 31.6% (*n* = 49) in participants of rural areas and 5.5% (*n* = 19) of urban area which was a finding (*p* < 0.0001).

**Table 2:** Association of demographic and epidemiological variables for seroprevalence of *anti-Brucella* antibodies in tested patients from Abbottabad, Pakistan based on Chi-square analysis.

**Variables**	**Total**	**Seropositive**	**Prevalence**	**Chi-square**	*P*-value^*^
	**participants**		**(%)**		
**Age (Years)**					
<25	131	16	12.2	1.01	0.6035
25–50	345	50	14.5		
>50	24	2	8.3		
**Gender**					
Male	199	24	12.1	0.47	0.493
Female	301	44	14.6		
**Urbanicity**					
Urban	345	19	5.5	59.83	0.0001
Rural	155	49	31.6		
**Animals Own/In House**					
Yes	122	39	31.9	44.29	0.0001
No	378	29	7.7		
**Animal Contact**					
Yes	162	50	30.9	58.63	0.0001
No	338	18	5.3		
**Processing/Handling Raw Animal Product/Meat**					
Yes	155	33	21.3	10.38	0.0013
No	345	35	10.1		
**Consuming Raw Animal Product**					
Yes	117	28	23.9	12.75	0.0004
No	383	40	10.4		
**Livestock Access to Source of Drinking Water**					
Yes	73	25	34.2	28.99	0.0001
No	427	43	10.1		
**Contact With Aborted Animals**					
Yes	33	13	39.4	17.72	0.0001
No	467	55	11.8		
**Brucellosis Related Symptoms in Any Other Family Member**					
Yes	81	21	25.9	11.28	0.0008
No	419	47	11.2		
**Pregnancy Status in Females**					
Yes	131	13	9.9	3.46	0.0629
No	170	31	18.2		
**Any Abortion History**					
Yes	21	5	23.8	3.2	0.0544
No	110	8	7.2		

*Chi-square test was applied*.

* *p-value < 0.05 were considered statistically significant*.

Several risk factors that related with the spread of brucellosis from animals to humans were determined (Tables 2, 3). About 31.9% (*n* = 39) seropositive participants kept animals (cattle, goats, sheep, etc.) at their homes showing statistically significant relationship (*p* = 0.0001). The highest prevalence (30.9%; *n* = 50) was observed among participants who had direct contact with livestock (*p* = 0.0001). Processing or handling of raw animal products such as meat or milk etc. was also an important and significant factor (*p* = 0.0013) recorded for 33 (21.3%) patients. Consuming raw products of animals such as undercooked meat or unpasteurized milk was recorded for 28 patients (23.9%) and was found significant (*p* = 0.0004). 25 (34.2%) participants of the study reported that livestock had access to the source of their drinking water which was a significant finding (*p* = 0.0001). 13 (39.4%) participants had contact with material of aborted animals which was a significant finding (*p* = 0.0001). It was found that 21 (25.9%) of the patients had family members that had similar symptoms of brucellosis (*p* = 0.0008).

**Table 3:** Logistic regression analysis to determine odds ratio, 95% confidence interval, and *p*-value between brucellosis positive cases.

**Variables**	**OR**	**95% CI**	**DF**	***p*-Value**
Gender	0.801	0.47–1.36	1	0.413
Area (Rural, Urban)	0.126	0.07–0.22	1	0.0001
Animals in house	5.65	3.30–9.67	1	0.0001
Animal contact	7.93	4.44–14.17	1	0.0001
Processing raw animal product	2.39	1.42–4.02	1	0.0007
Consuming raw animal product	2.69	1.57–4.61	1	0.0002
Livestock access to source of drinking water	4.65	2.61–8.28	1	0.0001
Contact with aborted animals	4.86	2.29–10.33	1	0.0001
Brucellosis related symptoms in any other family member	2.77	1.54–4.95	1	0.0004
Pregnant status in females	0.494	0.24–0.98	1	0.0428
Abortion history in pregnant females	3.98	1.15–13.70	1	0.0356

Thirteen pregnant women were positive for brucellosis. The data analyzed were statistically non-significant between pregnant and non-pregnant women (*p* = 0.0629). Five (23.8%) SAT positive pregnant women had also an abortion history (*p* = 0.0544).

The most common clinical signs observed in positive patients were fever 94.1% (*n* = 64), arthralgia 55.8% (*n* = 38), generalized ache 55.1% (*n* = 34), anorexia 47% (*n* = 32), headache (32.3%) (*n* = 22), fatigue 32.3% (*n* = 22), nausea 26.4% (*n* = 18) and the least common clinical sign observed was night sweat 25% (*n* = 17). Similarly, the ratio of clinical signs observed in patients confirmed by RT-PCR (57) were fever 96.4% (*n* = 55), arthralgia 63.1% (*n* = 36), generalized ache 57.8% (*n* = 33), anorexia 52.6% (*n* = 30), head ache 36.8% (*n* = 21), fatigue 33.3% (*n* = 19), nausea 28.0% (*n* = 16), and the least common clinical sign observed was night sweat 26.3% (*n* = 15). The clinical symptoms observed in study participants are shown in Table 4.

**Table 4:** Clinical signs and symptoms of brucellosis in seropositive patients.

**Clinical presentations**	**SAT positive (*n* = 68)**	**Prevalence (%)**	**Real-time PCR positive (*n* = 57)**	**Prevalence (%)**
Fever	64	94.1	55	96.4
Night Sweats	17	25.0	15	26.3
Headache	22	32.3	21	36.8
Arthralgia	38	55.8	36	63.1
Generalized ache	34	55.1	33	57.8
Nausea	18	26.4	16	28.0
Anorexia	32	47.0	30	52.6
Fatigue	22	32.3	19	33.3

## Discussion

Brucellosis is a zoonotic disease of worldwide distribution. It negatively impacts human health, animal production and economy by significant loss (29). Human disease is directly related to animal brucellosis in farmed bovine and small ruminants and several risky behaviors such as consumption of unpasteurized dairy products and fail to use protective clothing during handling of potentially infectious animals and their products (30). Brucellosis patients are regularly misdiagnosed and mistreated due to lack of awareness of attending doctors and reliable laboratory diagnostic support. Thus, brucellosis may become chronic causing severe osteoarticular, cardiovascular, neurological, and genitourinary complications including epididymo-orchitis and abortion in pregnant women if left untreated (31, 32). Consequently, it is of prominent importance to diagnose brucellosis as early as possible and set on adequate therapy. Brucellosis is also one of the most frequent infective causes of fever of unknown origin in endemic regions (21, 22). Physicians working at outdoor patient departments of hospitals in Pakistan are aware of this fact nowadays and have slide SAT based on brucellosis febrile antigens and PCR as cheap and fast diagnostic tools at hand. This study was made to assess the usefulness of these tests at the local settings of Abbottabad and to identify risk factors that alert attending doctors to consider acute brucellosis in cases of fever of unknown origin (FUO).

Sixty eight (13.6%) out of 500 patients presenting with signs of acute illness at outdoor departments of three hospitals in Abbottabad, KPK were positive for anti-*Brucella* antibodies using SAT in this study. This prevalence shows the high burden of disease in the local population. This prevalence is in the expected range to be found in patients with FUO (21, 22). A recent study conducted in hospitals of Abbottabad found 70% of patients SAT positive (33). It has to be stressed that data from different studies cannot be compared without caution as various not standardized or harmonized tests are still used. Hence, these data show that physicians at hospitals in endemic areas should be aware of brucellosis in their day-to-day work.

Real-time PCR was performed on seropositive patient samples (*n* = 68) to assess its value for the diagnosis of acute brucellosis. Real-time PCR is a rapid, reliable, highly sensitive and specific method for molecular diagnosis but depends on the availability of specific DNA in the serum sample. In a recent study, *Brucella* was detected using real-time PCR from 24 serum samples of patients from six hospitals of Lahore Punjab Pakistan (7). In our study, 57 samples of presumably acute brucellosis patients tested positive, 96.4% of them presented with febrile illness at the day of admission. This result shows that genus specific real-time PCR is a rapid method to confirm brucellosis in FUO patients in endemic areas and reducing the risk of infection of laboratory personnel connected to culture.

Identifying risk factors for disease in a patient can help the attending doctor to choose the diagnostic means and to start a well-timed onset of the appropriate therapy then. The prevalence of brucellosis was highest (14.5%) in the age group 25–50 years in present study. This finding can be explained by the fact that participants of this middle-aged group were mainly veterinarians, butchers and milking personnel who were in close contact to animals. However, seropositive cases of brucellosis were reported in participants of all age groups. These findings are in accordance to those of other studies. Ali et al. found the highest prevalence in the age group of 20–30 years (26.92%) while a low prevalence was recorded in the age group >40 years (7.80%). This study was also done in the Punjab, Pakistan but RBPT and ELISA were used (19). A study was conducted in Southern Saudi Arabia to detect anti *Brucella* antibodies in febrile patients using SAT (34). Those researchers determined the highest seroprevalence in patients 21 and 40 years of age (35.8–45.3%), while low prevalence was recorded in young children and older people (3 and 15%), respectively. These authors want to stress that a comparison of data should be done with great care as different study designs and tests were used. Hence, an interpretation of the data points to the fact, that in rural populations the presence of antibodies is linked to the contact to brucellae or their LPS and with growing age it is very likely to find more positives. Neither anti-*Brucella* antibodies are detected in the study participants nor the hospitals give the final statement that an active infection caused rise in the level of these antibodies due to the discussed shortcomings of brucellosis serology. The interpretation of these data has to be done in the light of the epidemiological context. Trends, however, are obvious and can be used to guide countermeasures.

*Brucella* infection was found more often in women (14.6%) than in man (12.1%). Similarly, a higher prevalence of brucellosis in female patients (37.06%) was also recorded from hospitals of Peshawar, KPK using SPAT (6). In another study in KPK, serum samples from patients of pyrexia of unknown origin and febrile illness were tested by SAT and the prevalence of *Brucella* antibodies were higher in women (9.4%) than in man (5.4%) (12).

The explanation is that animal husbandry is done mainly by women in Pakistan. Therefore, they are in direct contact to animals during their daily activities and help during parturition without using precautionary measures. In contrast, all RBPT and standard tube agglutination test (STAT) positive patients from Ludhiana, India were reported to be man. In contrast to Pakistan, only few women were involved in activities that exposed them to animals and other potential risk-factors (35). Similarly, a study conducted in high-risk group persons from Bangladesh also found a higher prevalence in man (5.6%) than in women (0.8%) using SAT and ELISA. The main reason for this finding was, that mainly butchers, milkers, livestock farmers and veterinary practitioners were tested. These occupations are traditionally in the hands of men in Bangladesh and expose them to a high risk of infection (36). This is also true for the Egyptian setting where more men are involved in management of livestock (37).

This study found a higher prevalence in persons from rural areas than in people of the urban area. This finding is similar to that of a study conducted in Peshawar among hospital patients using SPAT (6). Persons from rural areas are often involved in birthing and herding of livestock as they are more dependent on livestock production putting them at the risk of infection (19). A cross sectional study was conducted on rural population of the Punjab in India and RBPT and ELISA seropositivity was also linked to a history of assisting with abortions and calving (38).

Nicoletti stated that each case of brucellosis in humans is related to an animal source and its presence in animals causes a major risk of *Brucella* infection for humans (39). Thus, animal contacts, processing or handling raw animal products or meat and consumption of raw foods are the main risk factors to be considered (40–42). Additionally, the risk factor “access of livestock to source of drinking water” was considered in this study to take into account the local epidemiological circumstances. In our setting *Brucella* positive animals can be a source of water contamination and these brucellae may survive for 28–113 days in tap water (43, 44). Indeed, this study showed the highest seroprevalence in the group of persons who had direct contact with livestock (30.9%) or raised livestock at home (31.9%). As expected, processing or handling raw animal foods proved to be an important risk factor (21.3%). Twenty-eight patients recorded consumption of undercooked meat or unpasteurized milk. 13 (39.4%) participants had direct contact with materials of aborted animals. Aborted fetuses are usually left for decomposition by scavengers (i.e., jackals, vultures, crows, cockroaches, etc.) instead of proper disposing. This procedure increases the infection risk because large numbers of organisms are excreted with the uterine fluid, placenta, and fetus at the calving/lambing time (45).

Brucellosis in pregnant women bares the risk of miscarriage and may also cause repeatedly abortions after becoming chronic. Thus, participants of this study were asked about the course of previous pregnancies. Indeed, out of 21 women with abortion history, 5 (23.8%) were seropositive. A recent study in Pakistan involving 429 pregnant women mostly from rural areas reported 5.8% seroprevalence using RBPT and 14.6% of these had abortion history (15). Due to the low number of cases in both studies, we only can recommend further studies to evaluate these findings because brucellosis can pose a serious risk to newborns. Mortality in newborns was reported as well as transmission of brucellosis to a neonate via the congenital route or via breastmilk. Future studies in Pakistan should also consider this neglected aspect of brucellosis in childhood (46–48).

The most common clinical sign observed among SAT seropositive patients was fever i.e., 94.1% (*n* = 64), followed by arthralgia with 55.8% (*n* = 38). Similarly, patients from hospitals of Peshawar, KPK reported pain, fever, insomnia, malaise, and aches (32). 30.8% (*n* = 21) positive participants of this study reported persons with similar symptoms of brucellosis in their households. Transmission from person to person is rare, so these household members were most probably infected by the same animals or foods. Brucellosis in endemic countries is a family problem. Physicians should be aware of that fact and include all family members in their investigations. As brucellosis can be attracted again and again from the same source it is of imminent importance to identify and eliminate this source as well. Patients must be made aware of the epidemiology of brucellosis and local veterinary officers need to be involved finally.

The sensitivity and specificity of diagnostic tests is very important. In general, the sensitivity and specificity of brucellosis serological tests vary in the literature also due to different cut-off values and unclear sample status. Sensitivity and specificity also depend on the characteristics of the population under study and the local epidemiological conditions (49). Studies in patients with clinical symptoms of brucellosis have shown that SAT is in principle well-suited for the detection of infection (50, 51). Combination of at least one of the conventionally used serological tests (e.g., RBPT, SAT, ELISA) with PCR was recommended for developing countries. In this way, the advantages of serological testing in terms of sufficiently high sensitivity can be combined with the high specificity of a PCR reaction (52). Furthermore, several samples were SAT positive, but were negative by qPCR, indicating past brucellosis. Therefore, qPCR may be capable of distinguishing between past and present infections.

## Conclusions

The present study reported the prevalence of brucellosis among patients including pregnant women who visited the outdoor patient departments from three hospitals of Abbottabad, Pakistan. The study showed that the population of Abbottabad is at risk of acquiring brucellosis because most people living in rural areas have close contact with animals and consume raw products of animals e.g., unpasteurized milk. The slide SAT based on febrile *Brucella* antigens can identify acute cases of brucellosis as shown by accompanying positive RT-PCR results. *Brucella* genus specific RT-PCR is a promising tool to diagnose brucellosis in febrile FUO patients. Hence, further research is needed to validate the results of this preliminary study. But the results of this study can already contribute to develop strategies for controlling human brucellosis in rural settings of Pakistan, to raise awareness about brucellosis in livestock professionals, consumers, and physicians and to develop control programs by authority in charge.

## Author's Note

LH is postgraduate student in Department of Microbiology, University of Haripur, Pakistan. SA is Assistant Professor in Discipline of Zoology, Department of Wildlife & Ecology, University of Veterinary and Animal Sciences, Lahore, Ravi Campus, Pattoki, Pakistan. He is group leader of One Health Research Lab. He is Principal Investigator of German Federal Foreign Office, project funded by “Building a network of laboratories in Pakistan to enhance biosafety and biosecurity in Pakistan.” MS is Chairman and Associate Professor of Department of Microbiology, The University of Haripur, Haripur, Pakistan. AH and SAA are clinical scientists from Department of Microbiology, Fauji Foundation Hospital, Rawalpindi, Pakistan. ST is Assistant Professor from Department of Zoology, Hazara University Mansehra, Pakistan. US is postgraduate student Discipline of Zoology, Department of Wildlife & Ecology, University of Veterinary and Animal Sciences, Lahore, Ravi Campus, Pattoki, Pakistan. FM is Head of NRL Animal Brucellosis at Institute of Bacterial Infections and Zoonoses, Friedrich-Loeffler-Institut, Naumburger Str. 96a, 07743 Jena, Germany. AK is an Assistant Professor at Department of Pathobiology, College of Veterinary and Animal Sciences, Jhang, Pakistan. HE-A is head of NRL Campylobacteriosis at Institute of Bacterial Infections and Zoonoses, Friedrich-Loeffler-Institut, Naumburger Str. 96a, 07743 Jena, Germany. HN is Head of Institute of Bacterial Infections and Zoonoses, Friedrich-Loeffler-Institut, Naumburger Str. 96a, 07743 Jena, Germany. He is Expert to the OIE for Brucellosis and Glanders.

## Data Availability Statement

The raw data supporting the conclusions of this article will be made available by the authors, without undue reservation.

## Ethics Statement

The studies involving human participants were reviewed and approved by Ethical Committee of the University of Haripur, Haripur, Pakistan (Approval Number: F. No (01) ORIC-UOH//2020/). The patients/participants provided their written informed consent to participate in this study.

## Author Contributions

LH, SA, MS, FM, HE-A, and HN conceptualized the study and did the manuscript write-up. LH, ST, US, AS, and SAA analyzed the data. LH, SA, MS, US, and AK wrote the article. All authors read and approved the final manuscript.

## Funding

This research was funded by German Federal Foreign Office, funded project Building a network of laboratories in Pakistan to enhance biosafety and biosecurity in Pakistan Grant Number: AA-OR12-370:43 BIOS FLIPAK.

## Conflict of Interest

The authors declare that the research was conducted in the absence of any commercial or financial relationships that could be construed as a potential conflict of interest.

## Publisher's Note

All claims expressed in this article are solely those of the authors and do not necessarily represent those of their affiliated organizations, or those of the publisher, the editors and the reviewers. Any product that may be evaluated in this article, or claim that may be made by its manufacturer, is not guaranteed or endorsed by the publisher.

## Abbreviations

SAT: serum agglutination test
BCSP-31: *Brucella* cell-surface protein 31
RT-PCR: real-time polymerase chain reaction
DNA: deoxyribonucleic acid
OIE: Office for International des Épizooties
DHQ: District Headquarters
KPK: Khyber Pakhtunkhwa
OPD: outdoor patient departments.
