# Supplementary material for: Risk Factors for Acute Brucellosis in Patients on the Day of Admission at Selected Hospitals of Abbottabad, Pakistan
Source: Front Public Health. 2022 Jan 31;9:669278. doi: 10.3389/fpubh.2021.669278 (PMC8841874; doi:10.3389/fpubh.2021.669278)
Supplement: Supplementary file 1 [file Table_1.docx]

**Table S1: Comparison of Slide Serum Agglutination Test and *Brucella* Genus Specific real-time PCR**

| **Sample Number** | **Gender** | **SAT** | ***Brucella* Genus Specific PCR** |
| --- | --- | --- | --- |
| 8 | Female | + | + |
| 11 | Male | + | + |
| 21 | Female | + | - |
| 23 | Male | + | + |
| 25 | Female | + | + |
| 26 | Male | + | - |
| 29 | Male | + | + |
| 33-34 | Female | + | + |
| 39 | Female | + | + |
| 44 | Female | + | + |
| 52 | Male | + | + |
| 61 | Female | + | + |
| 75 | Male | + | + |
| 86 | Female | + | + |
| 94 | Male | + | + |
| 104 | Female | + | + |
| 112 | Female | + | + |
| 124 | Female | + | + |
| 126 | Male | + | + |
| 135 | Male | + | + |
| 136 | Female | + | + |
| 148 | Female | + | + |
| 158 | Female | + | - |
| 165 | Female | + | + |
| 174 | Female | + | + |
| 175 | Male | + | + |
| 187 | Female | + | + |
| 194 | Female | + | + |
| 197 | Female | + | + |
| 205 | Male | + | + |
| 206 | Female | + | + |
| 218 | Female | + | + |
| 224 | Male | + | - |
| 227 | Female | + | + |
| 235 | Female | + | + |
| 243 | Female | + | - |
| 251 | Female | + | + |
| 254 | Male | + | + |
| 266 | Female | + | + |
| 274 | Female | + | - |
| 286 | Male | + | + |
| 293 | Female | + | + |
| 301 | Female | + | + |
| 305 | Male | + | + |
| 312 | Female | + | + |
| 324 | Male | + | + |
| 332 | Female | + | - |
| 340 | Female | + | + |
| 348 | Male | + | + |
| 351 | Female | + | + |
| 361 | Female | + | + |
| 371 | Male | + | + |
| 379 | Male | + | - |
| 390 | Male | + | + |
| 393 | Female | + | + |
| 403 | Female | + | - |
| 411 | Male | + | + |
| 417 | Female | + | + |
| 426 | Female | + | + |
| 435 | Male | + | + |
| 439 | Female | + | + |
| 440 | Female | + | + |
| 457 | Male | + | - |
| 467 | Female | + | + |
| 480 | Male | + | + |
| 493 | Female | + | - |
| 500 | Female | + | + |
